# Extraction of Individual EEG Gamma Frequencies from the Responses to Click-Based Chirp-Modulated Sounds

**DOI:** 10.3390/s23052826

**Published:** 2023-03-04

**Authors:** Aurimas Mockevičius, Yusuke Yokota, Povilas Tarailis, Hatsunori Hasegawa, Yasushi Naruse, Inga Griškova-Bulanova

**Affiliations:** 1 Institute of Biosciences, Life Sciences Centre, Vilnius University, Saulėtekio av. 7, LT-10257 Vilnius, Lithuania; 2 Center for Information and Neural Networks (CiNet), National Institute of Information and Communications Technology, Saka University, Kobe 651-2492, Hyogo, Japan

**Keywords:** individual gamma frequency (IGF), auditory steady-state response (ASSR), dry electrodes

## Abstract

Activity in the gamma range is related to many sensory and cognitive processes that are impaired in neuropsychiatric conditions. Therefore, individualized measures of gamma-band activity are considered to be potential markers that reflect the state of networks within the brain. Relatively little has been studied in respect of the individual gamma frequency (IGF) parameter. The methodology for determining the IGF is not well established. In the present work, we tested the extraction of IGFs from electroencephalogram (EEG) data in two datasets where subjects received auditory stimulation consisting of clicks with varying inter-click periods, covering a 30–60 Hz range: in 80 young subjects EEG was recorded with 64 gel-based electrodes; in 33 young subjects, EEG was recorded using three active dry electrodes. IGFs were extracted from either fifteen or three electrodes in frontocentral regions by estimating the individual-specific frequency that most consistently exhibited high phase locking during the stimulation. The method showed overall high reliability of extracted IGFs for all extraction approaches; however, averaging over channels resulted in somewhat higher reliability scores. This work demonstrates that the estimation of individual gamma frequency is possible using a limited number of both the gel and dry electrodes from responses to click-based chirp-modulated sounds.

## 1. Introduction

A great interest in individualized markers of brain activity that have potential clinical or neuro-technological applications has recently emerged. This attention has largely been drawn to electroencephalography (EEG), which provides cheap and fast assessment opportunities which are applicable even outside the laboratory in ecologically valid settings. The analysis of the signal offers large possibilities with a focus on versatile domains and functional outcomes. Several authors have addressed individual resonant frequencies, i.e., the largest frequencies of the activity of subjects, as a reflection of the state of neural networks relating them to certain functional manifestations. To illustrate, peak alpha frequencies have been shown to be related to performance in cognitive tasks [1,2], whereas peak theta frequencies were proposed to relate to cognitive control [3]. Similarly, peak frequencies in the gamma range were addressed. The gamma-range activity has been argued to be important for many cognitive and sensory processes and is frequently impaired in neuropsychiatric conditions. For example, the preferred frequencies in the gamma range were related to the ability to detect a gap in the sounds, i.e., to the temporal sampling rate in the auditory system [4,5]. Additionally, peak frequencies in the gamma range were shown to decline with age [6,7] and to “slow down” in subjects with developmental dyslexia [8], patients with schizophrenia [9,10], or Alzheimer’s disease [6]. This suggests that peak gamma frequencies might have a physiological meaning and deserve further investigation.

However, since a prominent peak in the gamma activity is usually not observed in the EEG frequency spectra, determining individual-specific dominant gamma frequency (individual gamma frequency, IGF) is not a straightforward task. It is not entirely clear what is the best method of measurement for gamma range preferred frequencies. Attempts were made to extract it from resting-state EEG data [6], from a response to transient sensory stimuli [11,12], or in response to some meaningful cognitive tasks and related events [13,14]. Alternatively, the periodic stimulation testing of the most preferred frequency, defined as generating the largest response, was employed utilizing an auditory steady-state approach. To illustrate, Zaehle et al. stimulated using amplitude-modulate sounds at single frequencies spanning a 20–100 Hz range and estimated the preferred gamma frequencies to be around 30–60 Hz with a peak at 48 Hz [15]. Similarly, Gransier et al. tested a range of between 0.5 and 100 Hz showing that peak was within the 30–60 Hz range, with a mean of 45 Hz [16]. However, stimulation with single frequencies is time-consuming and problematic for clinical assessment; thus more elaborate approaches need to be developed. As an alternative, a chirp-based stimulation was proposed, demonstrating its capability to detect peak responses in the gamma range [17,18]. Chirp sounds represent a stimulation type where the amplitude modulation of the carrier covers certain frequency ranges of interest. However, amplitude-modulated sounds are known to evoke less pronounced EEG responses [19]. To utilize the benefits of the click-based stimulation that produces strong brain responses, we recently tested the ability of stimuli composed of single clicks when spaced in a logarithmic manner to evoke gamma-range responses [20]. This approach demonstrated that in response to stimulation, a peak in the gamma range could be observed, and responses at the peak were related to certain cognitive abilities, namely, the time needed to perform complex information-processing tasks [20,21].

The abovementioned works were performed in laboratory settings using research-grade EEG equipment. Nevertheless, modern experimental situations require that the methods work in less controlled experimental settings, e.g., on the data of a small number of dry EEG channels that allow for fast assessment. This would enable easier translational application and assessment in more naturalistic settings.

In this work, we tested whether it was possible to reliably extract individual gamma peak information from the responses to auditory chirp-based stimulations collected with research-grade EEG equipment and dense electrode placement over the region of interest where a response was observed. Then, we tested the approach on data collected with custom-made dry EEG electrodes and a low number of EEG channels. We focused on the estimation of IGF based on the phase-locking measure that was shown to produce the strongest and most reliable results for classical auditory-steady state responses [22] and more pronounced results for click-based chirp stimulation [20,21].

## 2. Materials and Methods

### 2.1. Participants

A group of 80 young participants (42 females, 2 left-handed; mean age ± SD: 26.07 ± 4.28) without a reported history of psychiatric and neurological disorders participated in the study using a high-density EEG system. The hearing thresholds of all the subjects were within the normal range (<25 dB HL at octave frequencies). Participants abstained from alcohol 24 h prior to the testing and did not consume nicotine and caffeine-containing drinks for at least one hour prior to the experiment. The study was approved by the Vilnius Regional Biomedical Research Ethics Committee (no. 2020/3-1213-701), and all participants provided their written informed consent.

A group of 33 young subjects (15 females; mean age ± SD: 27.8 ± 5.85) without a reported history of psychiatric and neurological disorders participated in this study utilizing a custom-made dry electrode EEG system. All subjects had normal hearing along with normal or corrected-to-normal vision. Subjects provided written informed consent after the procedural details had been explained and before the experiment. All experimental procedures were approved by the Ethics Committee for Human and Animal Research of the National Institute of Information and Communications Technology (no. B210152204). The experiment was performed in accordance with the ethical standards described in the Declaration of Helsinki.

### 2.2. EEG Acquisition

A 64-channel EEG signal was recorded with an ANT device (ANT Neuro, Hengelo, The Netherlands) and WaveGuard EEG gel-based cap with integrated Ag/AgCl electrodes which were placed according to the 10-10 International electrode placement system. Mastoids were used as a reference; the ground electrode was attached close to Fz. Impedance was kept below 20 kΩ, and the sampling rate was set at 1024 Hz. Simultaneously, vertical and horizontal electro-occulograms (VEOG and HEOG) were recorded from above and below the left eye and from the right and left outer canthi.

The 3-channel EEG data were collected using a wireless portable system (PolymateMini AP108, Miyuki Giken Co., Ltd., Tokyo, Japan) with three active dry electrodes (Unique Medical Co., Ltd., Tokyo, Japan) [23] positioned at FC3, FCz, and FC4 according to the 10–20 International electrode placement system. The right mastoid was used as a reference; the ground electrode was attached to the left mastoid. The sampling rate was set at 500 Hz. Simultaneously, vertical and horizontal electro-occulograms (VEOG and HEOG) were recorded from above and at the side of the left eye.

### 2.3. Auditory Stimulation

**Figure 1:**
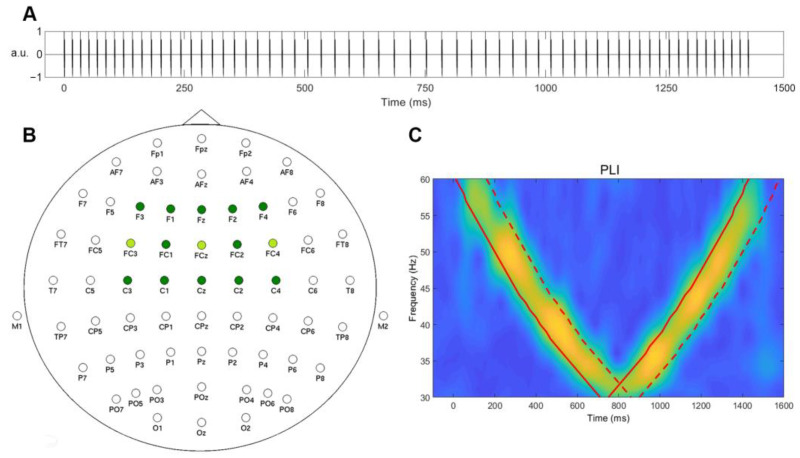
(**A**) A schematic representation of the sound stimulus used in this study. (**B**) Electrode placement for 64- and 3-channel systems. Channels used for analysis are colored in green. (**C**) A schematic representation of time-window definition for the calculation of IGFs from PLI. The bold red line indicates the timing of the stimulation; the red dashed line denotes the edge of averaging window (+150 ms). a.u.—arbitrary units.

Stimulus trains were created of single identical 1.5 ms white-noise bursts of alternating polarity spaced with changing inter-click periods to cover a range from 30 to 60 Hz in a decreasing-then-increasing order. The duration of the stimulus train was 1500 ms, and 200 repetitions were presented with 700–1000 ms inter-stimulus intervals. The schematic representation of the sounds used is presented in Figure 1A. The auditory stimuli were designed in the Matlab 2014 environment (The MathWorks, Inc., Natick, MA, USA) and presented binaurally through Shure SE215 earphones (in the 64-channel group) and through RHA MA750 earphones (in three dry electrode groups). The sound pressure level was set at 60 dB.

### 2.4. EEG Processing

The 64-channel EEG data were pre-processed in EEGLAB for MatLab© [24] in a manner as described in previous research [20]. The power-line noise was removed using multi-tapering and Thomas F-statistics (CleanLine plugin for EEGLAB). The data were visually inspected, and channels with substantial noise (shift, movements) were removed. Further, EEG data were submitted to an independent component analysis (ICA) that was performed with the ICA-implementation of EEGLAB (‘runica’ with default settings [25,26]) after Independent components relating to eye movements (blinks and saccades), and ECG were removed. The removed channels were then reconstructed using a 3D spherical spline method [27].

The 3-channel EEG data were offline pre-processed in EEGLAB for MatLab© [24]. An ICA was performed with the ICA implementation of EEGLAB (‘runica’ with default settings) after the visual inspection. Independent components related to eye movements (blinks and saccades) were removed.

### 2.5. Individual Gamma Frequency Extraction

The analysis of all the data was run using Fieldtrip [28] functions in MATLAB R2020a. Time-frequency transformation using a complex Morlet wavelet (14 cycles) was applied to the signal within a 1–120 Hz range. The phase-locking index (PLI) was used as a measure of interest and was known to be least sensitive to noise and produced the most stable results. To create responses for each subject, 100 iterations were run with 100 randomly selected epochs. In the 64-channel group, electrodes covering the frontocentral region where a gamma response to auditory stimulations was consistently observed and selected for the analysis (Figure 1B). For the 3-channel data, all electrodes were included in the analysis. The responses were averaged within 150 ms time intervals for each frequency from 30 to 60 Hz, in steps of 1 Hz. The averaging windows (marked with a red dashed line in Figure 1C) were selected based on the time onset of the corresponding frequency in the chirp-like stimulus (red bold line in Figure 1C), both in the chirp-down and chirp-up periods (Figure 1C). 

Several IGF estimation approaches were tested. First, different sets of channels were selected for 64-channel data: 15 channels (F3, F1, Fz, F2, F4, FC3, FC1, FCz, FC2, FC4, C3, C1, Cz, C2, and C4) or 3 channels (FC3, FCz, and FC4). Secondly, for both sets of selected channels, the PLI values in chirp-down and chirp-up periods were either averaged together to obtain a single IGF estimate for each frequency or were analyzed separately to obtain two IGF estimates–one for the down part and one for the up part. This was conducted in order to account for the possibility that “slowing” or “speeding” (frequency change) could depend on the direction of stimulation. These 6 approaches are further referred to as “IGF extraction condition”: electrodes kept, down-up; electrodes averaged, down-up; electrodes kept, down; electrodes kept, up; electrodes averaged, down; electrodes averaged, up. Furthermore, the outputs within each selected channel were also averaged or kept separated. In all of these approaches, 5 dominant frequencies within a 30–60 Hz range with the highest PLI values were extracted for each trial iteration (and channel, if channels were not averaged), resulting in a trial iteration (×channel) × the top 5 frequencies of the matrix for each subject. 

**Figure 2:**
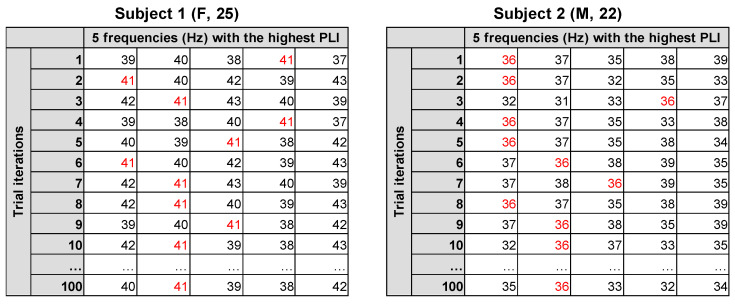
An example of IGF estimation on an average of 15 channels and averaged chirp-down and chirp-up parts in two subjects: the matrix of 100 trial iterations and 5 frequencies displaying the highest PLI response. The extracted IGF is marked in red.

To estimate the most prevalent IGFs, the mode was computed from all the values within the matrix for each subject following the reasoning of Bjekić et al. [29]. The participant-level reliability of IGF was calculated as the ratio between the number of IGF values within the whole matrix and the total number of cells within the matrix after excluding the last dimension, which represented the top 5 frequencies. The rationale behind choosing this divisor was that any frequency value could be present only once among a single set of the top 5 frequencies, thus excluding the last dimension, which allowed one to estimate how consistently the IGF value appeared among the dominant 5 frequencies in each trial iteration and (if not averaged) each channel. The computed IGF reliability ratios of all subjects were further divided into ranges: singular IGF (>0.8), high IGF reliability (0.51–0.8), medium IGF reliability (0.31–0.5), low IGF reliability (0.16–0.3), and no IGF (≤0.15). The example of IGF estimation from a single subject is presented in Figure 2. To further compare the reliability ratios across different IGF extraction conditions, a non-parametric Friedman test and post hoc Wilcoxon pairwise comparisons with Bonferroni correction were applied.

## 3. Results

**Figure 3:**
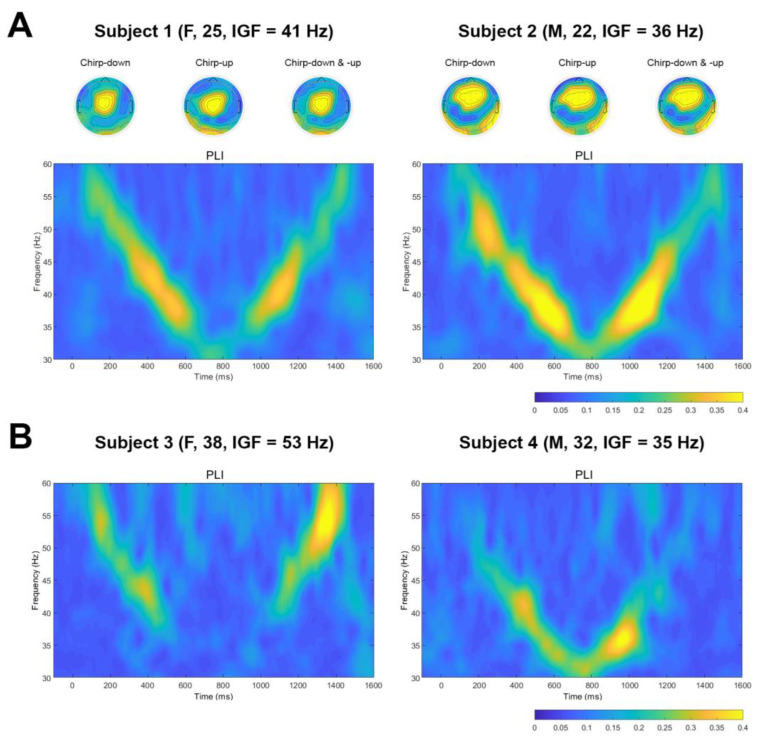
Example of time-frequency plots of PLIs. (**A**) Time-frequency plots of PLIs for two subjects from 15 gel electrodes data. Topoplots at IGF were created separately for chirp-down, chirp-up, and the averaging of both parts. (**B**) Time-frequency plots of PLIs for two subjects from three dry electrodes data.

For visualization purposes, the time-frequency plots of PLIs for two representative subjects of data averaged over 15 gel electrodes with corresponding topographies at estimated IGF for chirp-down, chirp-up, and both parts averaged (A), and time-frequency plots of PLIs for two representative subjects for data averaged over 3 dry electrodes (B) are presented in Figure 3.

### 3.1. 64-channel Gel Electrode System

The descriptive statistics of IGF estimation for all the tested conditions are presented in Table 1. Alongside the mean values, ranges of estimated IGF values and reliability scores for every method tested are presented.

#### 3.1.1. Chirp-Down and Up Averaged

The analysis on averaged chirp-down and chirp-up parts when each of the 15 channels was evaluated separately yielded the IGFs for each subject with a mean of 37 (±4) Hz and a reliability ratio of 0.67 (±0.16). The reliability scores mostly ranged from high to medium, with only one case of low reliability. When channels were averaged, the mean IGF was 37 (±4) Hz, and the reliability ratio was, on average, 0.89 (±0.12). The reliability scores ranged from a very high to high, with only one medium reliability case.

In the case of three channels, when analyzed separately, averaging chirp-down and chirp-up parts yielded IGFs of 37 (±4) Hz with a reliability ratio of 0.71 (±0.18). Reliability scores were mostly in a range from high to medium. The analysis of IGFs on chirp-up and down averaged parts when three channels were averaged estimated the IGFs to be 36 (±4) Hz, with a reliability of 0.88 (±0.14). The reliability scores were mostly very high or high. 

#### 3.1.2. Chirp-Down and Up Separate

The analysis on separate chirp-down and chirp-up parts and each of the 15 channels separately yielded comparable IGFs in chirp-down (37 ± 5 Hz) and chirp-up periods (37 ± 3 Hz); however, for the chirp-down period, the reliability ratio was somewhat lower (0.59 ± 0.16) than for chirp-up (0.66 ± 0.13). In both cases, reliability scores predominantly fell into a range from high to medium. Correlations between IGFs were calculated to see how IGF in chirp-down and up parts were related. A significant positive correlation was obtained (r = 0.47, *p* < 0.001). When IGFs were analyzed separately for chirp-down and chirp-up periods, with 15 averaged channels, IGF for the chirp-down period was 38 (±5) Hz, and for the chirp-up period was 37 (±3) Hz. The reliability ratio for chirp-down was slightly lower (0.83 ± 0.15) than for chirp-up (0.89 ± 0.13); however, in both cases, the reliability scores were in favor of either singular IGF or high-reliability outcome. Correlations between the IGFs confirmed that estimates from the chirp-down and chirp-up parts were positively related (r = 0.56, *p* < 0.001).

When chirp-down and chirp-up parts were analyzed separately on three electrodes, IGFs for the chirp-down period were at 37 (±5) Hz with a reliability ratio of 0.64 (±0.18), and for the chirp-up part at 37 (±4) Hz with a reliability of 0.69 (±0.16). The reliability scores were mostly in a range from high to medium. Correlations between IGF values for both periods revealed a significant positive association (IGF: r = 0.45, *p* < 0.001). When the three electrodes were averaged, and the chirp-down and chirp-up parts were analyzed separately, IGFs for the chirp-down period were estimated at 38 (±6) Hz with a reliability ratio of 0.82 (±0.16) and for the chirp-up parts at 37 (±4) Hz with the reliability ratio of 0.87 (±0.13). The reliability scores fell into a range from very high to medium. IGFs in chirp-down and chirp-up periods were positively correlated (IGF: r = 0.44, *p* < 0.001).

#### 3.1.3. Comparison of Reliability Ratios across IGF Extraction Conditions

There was a statistically significant difference in reliability ratios depending on the extraction condition (χ^2^(11) = 587.55, *p* < 0.001). Post hoc pairwise comparisons (Supplementary Material, Table S1) showed significant differences in reliability estimates between conditions with averaged electrodes vs. the electrodes kept, regardless of the number of channels and whether the chirp-down and chirp-up parts were taken together or separately. No difference in reliability ratios was observed between the chirp-down and chirp-up extraction conditions. In addition, significant differences were not present when comparing the reliability estimates from 15-channel and 3-channel extraction conditions.

### 3.2. 3-Channel Dry Electrode System

The descriptive statistics of IGF estimation in all the tested conditions are presented in Table 2. Alongside the mean values, ranges of estimated IGF values and reliability scores for every method tested are presented.

#### 3.2.1. Chirp-Down and Up Averaged

The analysis on averaged chirp-down and chirp-up parts when each of the three channels was evaluated separately yielded IGFs of 41 (±8) Hz with a reliability ratio of 0.71 (±0.18). Reliability scores were mostly defined in a range from high to medium. The analysis of IGFs on chirp-up and down parts together when the three channels were averaged estimated the IGFs to be 41 (±8) Hz, with a reliability of 0.75 (±0.17). The reliability scores were mostly very high and high. 

#### 3.2.2. Chirp-Down and Up Separate

When the chirp-down and chirp-up parts were analyzed separately on three electrodes, IGFs for the chirp-down period were at 42 (±10) Hz with a reliability ratio of 0.70 (±0.17), and for the chirp-up part at 41 (±7) Hz with a reliability of 0.72 (±0.18). The reliability scores mostly ranged from high to medium. Correlations between IGF values for both periods revealed a significant positive association (IGF: r = 0.53, *p* < 0.005). When chirp-down and chirp-up parts were analyzed separately on three averaged electrodes, the IGFs for the chirp-down period were estimated at 42 (±9) Hz with a reliability ratio of 0.75 (±0.17), and for the chirp-up parts at 40 (±7) Hz with the reliability ratio of 0.75 (±0.17). The reliability scores fell into a very high–medium range. IGFs in chirp-down and chirp-up periods were positively correlated (IGF: r = 0.60, *p* < 0.001).

#### 3.2.3. Comparison of Reliability Ratios across IGF Extraction Conditions

There was a statistically significant difference in reliability ratios depending on the extraction condition (χ^2^(5) = 22.07, *p* < 0.001). Post hoc pairwise comparisons (Supplementary Material, Table S2) showed significant differences in reliability estimates between the corresponding conditions with averaged electrodes vs. the electrodes kept. No differences in reliability ratios were observed between the chirp-down and chirp-up extraction conditions.

## 4. Discussion

Recently, attention has been drawn to the individualized parameters of the EEG signal, which could efficiently be used as biomarkers or as a guide to track brain activity for neurotechnological applications. One of the parameters is the individual gamma peak frequency (IGF), which has shown some promising physiologically relevant changes in clinical populations [8,10,30]. However, an efficient way for IGF estimation still needs to be developed. The analysis of periodic responses to periodic stimulation stands as one of the ways to probe brain oscillations [31]. This approach is frequently used in neuropsychiatric conditions, where the great potential of the responses was shown [32,33]. Several works demonstrated not only the gamma response per se but also the preferred frequency of the response to show physiologically meaningful changes [7,34], suggesting that this parameter should be investigated further as well.

This study tested the possibility of reliably extracting individual gamma peak information from the responses to auditory chirp-based stimulation collected with research-grade EEG equipment and dense electrode placement over the region of interest where a response was observed. The same approach was tested on the data collected with custom-made dry EEG electrodes and a low number of EEG channels. 

We showed that responses to auditory chirp-based stimulation could be recorded with both systems (Figure 3). Moreover, using chirp-based stimulation, we were able to reliably estimate the IGFs with both research-grade gel electrode and low-density dry electrode systems. According to the results (Table 1 and Table 2), the reliability scores obtained from the data recorded with gel electrodes for some IGF extraction conditions (e.g., “Electrodes averaged, down-up”, “Electrodes averaged, down”, and “Electrodes averaged, up”) were somewhat better than the data collected with dry electrodes (0.89 ± 0.12, 0.83 ± 0.15, 0.89 ± 0.13 for 15 gel electrodes and 0.88 ± 0.14, 0.82 ± 0.16, 0.87 ± 0.13 for 3 gel electrodes versus 0.75 ± 0.17, 0.75 ± 0.17 and 0.75 ± 0.17 for three dry electrodes). However, when electrodes were not averaged, and chirp-up and down parts (“Electrode kept, down” and “Electrode kept, up”) were assessed separately, the reliability of IGF estimates from the dry electrode system somewhat outperformed the gel electrode system (0.59 ± 0.16, 0.66 ± 0.13 for 15 gel electrodes and 0.64 ± 0.18, 0.69 ± 0.16 for 3 gel electrodes versus 0.70 ± 0.17, 0.72 ± 0.18 for three dry electrodes). The observed effect could partly be explained by the different signal-to-noise ratios (SNR) of the two systems. In general, the SNR of dry electrodes is low [35], and the extracted gamma from dry electrodes could have been overall less reliable due to the captured noise (including common phase noise), thus averaging had little effect on PLIs and reliability scores (all conditions close to 0.70–0.75, Table 2).

Importantly, our results showed that IGFs could be reliably estimated from three channels placed within the region of interest. In line with previous observations, the largest activation in response to auditory stimulation was evident in the frontocentral region (topoplots, Figure 3A), and that was very similar for various IGFs in both this study and previous reports [20,21]. This finding is also in line with earlier studies using responses to chirp stimulation and showing that even information from a single channel placed in the region of interest can provide physiologically relevant information [36,37]. Still, although no major difference in reliability scores obtained from data of 15 gel channels versus three gel channels could be observed (Table 1), averaging over channels and chirp-up and down parts contributed to somewhat better reliability estimates–this approach showed the best reliability scores for all conditions (fifteen gel channels, three gel channels, and three dry channels) that can be explained by increasing SNR [38].

We used chirp-down-up stimulation to take into account the fact that “slowing” or “speeding” could depend on the direction of stimulation (frequency change). As can be seen in Table 1 and Table 2, averaging over channels, in general, was slightly better for producing more reliable outcomes than the averaging of chirp-up and down parts. This potentially suggests that for IGF estimation, the stimulation duration could be reduced by keeping only chirp-up or down part, making the overall procedure faster and more comfortable for the subject. Previously, responses to the chirp-up and chirp-down stimuli were shown to not differ, and gamma-range activity did not depend on the attention level of the subject [39,40]. Moreover, IGFs estimated from chirp-down and chirp-up parts were significantly correlated in the current report (correlation coefficients ranged between 0.44 to 0.60), suggesting that IGFs could be extracted from the stimulation of any direction.

The proposed IGF extraction method can be easily implemented in research settings both from auditory stimulation and IGF extraction perspectives, even when only simple equipment with a low number of dry electrodes is available. The IGF estimation from responses to click-based chirps has been implemented in studies on healthy young participants by our group before [20,21] employing the simple maximal response detection approach. The method proposed in the current study is expected to produce more reliable results; however, it should further be tested in more diverse populations–older subjects or clinical groups–where changes in IGF could be physiologically meaningful.

## 5. Conclusions

The proposed approach to estimate individual gamma frequencies in response to the auditory click-based chirp stimulation resulted in the reliable estimation of IGFs using both the gel and dry electrode systems. The higher reliability of extracted IGFs was observed for data that were averaged over channels and chirp parts for the gel electrode system, and averaging over channels was more efficient for both the gel and dry electrode systems than averaging over chirp parts.

## Figures and Tables

**Table 1:** Descriptive statistics of the IGF estimations and IGF reliability intervals from 64-channel gel electrode data.

		Descriptive Statistics				Reliability Intervals *				
	IGF Extraction Condition	Mean IGF (Hz)	IGF Range (Hz)	Mean Reliability	Reliability Range	Singular IGF (*n*)	High (*n*)	Medium (*n*)	Low (*n*)	No IGF (*n*)
15 channels	Electrodes kept, down-up	37 (±4)	30–47	0.67 (±0.16)	0.27–0.98	15	47	17	1	0
	Electrodes averaged, down-up	37 (±4)	30–47	0.89 (±0.12)	0.47–1.0	62	17	1	0	0
	Electrodes kept, down	37 (±5)	31–53	0.59 (±0.16)	0.29–0.95	11	42	26	1	0
	Electrodes kept, up	37 (±3)	30–45	0.66 (±0.13)	0.32–0.97	10	59	11	0	0
	Electrodes averaged, down	38 (±5)	31–52	0.83 (±0.15)	0.51–1.0	47	33	0	0	0
	Electrodes averaged, up	37 (±3)	30–46	0.89 (±0.13)	0.58–1.0	62	18	0	0	0
3 channels	Electrodes kept, down-up	37 (±4)	30–49	0.71 (±0.18)	0.38–1.0	28	40	12	0	0
	Electrodes averaged, down-up	36 (±4)	30–49	0.88 (±0.14)	0.47–1.0	58	21	1	0	0
	Electrodes kept, down	37 (±5)	31–52	0.64 (±0.18)	0.34–0.98	18	41	21	0	0
	Electrodes kept, up	37 (±4)	30–50	0.69 (±0.16)	0.32–0.99	22	47	11	0	0
	Electrodes averaged, down	38 (±6)	30–52	0.82 (±0.16)	0.48–1.0	48	30	2	0	0
	Electrodes averaged, up	37 (±4)	30–51	0.87 (±0.13)	0.42–1.0	57	22	1	0	0

* Singular: >0.8; high reliability: 0.51–0.8; medium reliability: 0.31–0.5; low reliability: 0.16–0.3; no IGF: ≤0.15.

**Table 2:** Descriptive statistics of the IGF estimations and IGF reliability intervals from 3-channel dry electrode data.

		Descriptive Statistics				Reliability Intervals *				
	IGF Extraction Condition	Mean IGF (Hz)	IGF Range (Hz)	Mean Reliability	Reliability Range	Singular IGF (*n*)	High (*n*)	Medium (*n*)	Low (*n*)	No IGF (*n*)
3 channels	Electrodes kept, down-up	41 (±8)	31–57	0.71 (±0.18)	0.34–1.0	9	19	5	0	0
	Electrodes averaged, down-up	41 (±8)	31–57	0.75 (±0.17)	0.38–1.0	14	14	5	0	0
	Electrodes kept, down	42 (±10)	30–60	0.70 (±0.17)	0.33–0.99	10	19	4	0	0
	Electrodes kept, up	41 (±7)	30–59	0.72 (±0.18)	0.30–1.0	12	16	4	1	0
	Electrodes averaged, down	42 (±9)	30–60	0.75 (±0.17)	0.37–0.99	14	16	3	0	0
	Electrodes averaged, up	40 (±7)	30–60	0.75 (±0.17)	0.35–1.0	15	16	2	0	0

* Singular: >0.8; high reliability: 0.51–0.8; medium reliability: 0.31–0.5; low reliability: 0.16–0.3; no IGF: ≤0.15.

## Data Availability

The data presented in this study are available on request from the corresponding author. The data are not publicly available due to privacy restrictions.

## Supplementary Materials

The following supporting information can be downloaded at: https://www.mdpi.com/article/10.3390/s23052826/s1. Table S1: *p* values of Wilcoxon pairwise comparison across IGF extraction conditions, wet electrode data; Table S2: *p* values of Wilcoxon pairwise comparison across IGF extraction conditions, dry electrode data.
