# Supplementary material for: Extraction of Individual EEG Gamma Frequencies from the Responses to Click-Based Chirp-Modulated Sounds
Source: Sensors (Basel). 2023 Mar 4;23(5):2826. doi: 10.3390/s23052826 (PMC10007152; doi:10.3390/s23052826)
Supplement: Supplementary file 1 [file sensors-23-02826-s001.zip › sensors-2167260-supplementary.pdf]

Table S1. P values of Wilcoxon pairwise comparison across IGF extraction conditions with wet electrodes.

	(1)	(2)	(3)	(4)	(5)	(6)	(7)	(8)	(9)	(10)	(11)
(1) 15 electrodes kept, down-up	-										
(2) 15 electrodes kept, down	0.107	-									
(3) 15 electrodes kept, up	1.000	0.157	-								
(4) 3 electrodes kept, down-up	1.000	0.001	1.000	-							
(5) 3 electrodes kept, down	1.000	1.000	1.000	0.751	-						
(6) 3 electrodes kept, up	1.000	0.002	1.000	1.000	1.000	-					
(7) 15 electrodes averaged, down-up	< 0.001	< 0.001	< 0.001	< 0.001	< 0.001	< 0.001	-				
(8) 15 electrodes averaged, down	< 0.001	< 0.001	< 0.001	0.005	< 0.001	< 0.001	0.017	-			
(9) 15 electrodes averaged, up	< 0.001	< 0.001	< 0.001	< 0.001	< 0.001	< 0.001	1.000	0.177	-		
(10) 3 electrodes averaged, down-up	< 0.001	< 0.001	< 0.001	< 0.001	< 0.001	< 0.001	1.000	0.570	1.000	-	
(11) 3 electrodes averaged, down	< 0.001	< 0.001	< 0.001	0.009	< 0.001	< 0.001	0.013	1.000	0.122	0.435	-
(12) 3 electrodes averaged, up	< 0.001	< 0.001	< 0.001	< 0.001	< 0.001	< 0.001	1.000	1.000	1.000	1.000	1.000

Table S2. P values of Wilcoxon pairwise comparison across IGF extraction conditions with dry electrodes.

	(1)	(2)	(3)	(4)	(5)
(1) 3 electrodes kept, down-up	-			-	
(2) 3 electrodes kept, down	1.000	-			-
(3) 3 electrodes kept, up	1.000	1.000	-		
(4) 3 electrodes averaged, down-up	< 0.001	1.000	1.000	-	
(5) 3 electrodes averaged, down	1.000	< 0.001	1.000	1.000	-
(6) 3 electrodes averaged, up	1.000	1.000	< 0.001	1.000	1.000
